# Reproducibility of 3D scanning in the periorbital region

**DOI:** 10.1038/s41598-021-83335-5

**Published:** 2021-02-11

**Authors:** Maria H. J. Hollander, Joep Kraeima, Anne M. L. Meesters, Konstantina Delli, Arjan Vissink, Johan Jansma, Rutger H. Schepers

**Affiliations:** 1 Department of Oral and Maxillofacial Surgery, University Medical Center Groningen (UMCG), University of Groningen, PO Box 30.001, 9700 RB Groningen, The Netherlands; 2 Department of Trauma Surgery, University Medical Center Groningen (UMCG), University of Groningen, PO Box 30.001, 9700 RB Groningen, The Netherlands

**Keywords:** Health care, Medical research

## Abstract

The reproducibility of scanning in the periorbital region with 3D technology to enable objective evaluations of surgical treatment in the periorbital region was assessed. Facial 3D-scans of 15 volunteers were captured at different time points with a handheld Artec Space Spider structured light scanner. Two scans were made with a one minute interval and repeated after 1 year; for both a natural head position and with the head in a fixation-device. On assessing the area between the eyelashes and eyebrows, the medians of the average deviations between the various cross-sections of the one minute interval 3D-scans ranged from 0.17 to 0.21 mm at baseline, and from 0.10 to 0.11 mm when the minute-interval scanning was repeated one year later. The systematic differences when scanning in a natural head position and fixated position were comparable. The reproducibility of the 3D processing was excellent (intraclass correlation coefficient > 0.9). The repeated scanning deviations (baseline versus one year data) were well within the accepted clinical threshold of 1 mm. Scanning with a hand-held 3D-scanning device (Artec Space Spider) is a promising tool to assess changes in the periorbital region following surgical treatment since the median deviations are well below the clinically accepted 1 mm measuring error, for both the natural head and fixated positions.

## Introduction

Three-dimensional (3D) scanning is a practical method for objective visual comparisons of surgical results. To assess treatment outcomes accurately following facial surgery, pre- and post-treatment 3D-photographs must be captured with the same facial expression^1^. Since 3D-imaging is affected by changes in facial expression, muscle tone and head posture, it is of key importance to minimize such variability to obtain a reproducible 3D-scan. If the variation within the face in a resting position is large, the evaluation of the effect of surgery based on a pre- and postoperative 3D-photograph will be inaccurate, especially when only minor improvements are anticipated. Two main optical 3D-scanning technologies are commonly applied to evaluate human subjects, i.e. image-based scanners and range-based scanners^2–4^. Image-based scanners (stereophotogrammetry) reconstruct the surface geometry with the use of two or more photographic images taken from different positions and this creates a point cloud of 3D coordinates. Stereophotogrammetry is an accurate user friendly image-based scanner method for comparing 3D-photographs of the same individual at different time points^5–9^. For this reason, stereophotogrammetry has been used to evaluate postoperative changes in the soft tissues of the face following orthognathic surgery and cranio-maxillofacial surgery^7,10^. Range-based scanners, such as structured light scanners, project a series of linear patterns of light onto the object to scan and capture its reflection with a sensor. Trigonometric triangulation is used to calculate the reflection angle of the structured light, and the three-dimensional coordinates are used to digitally reconstruct the object.

Irrespective of the type of scanner applied, the obtained point clouds are mathematically fused to polygonal 3D-meshes.

Although the accuracy of the above mentioned 3D-systems have been validated^2,8,9,11–14^, scanning the periorbital region in a reproducible way remains challenging. Maal et al.^1^ found a mean overall variation of 0.25 mm within the face at rest with the largest variations occurring in the mouth and eye regions. This larger variation in the eye region may be explained by the difficulty in capturing the eyes correctly when using 3D stereophotogrammetry^5^. The Verhulst et al.^2^ study, which compared three different 3D-systems (3dMDface system, Vectra XT, Artec Eva), even excluded the periorbital area from the analysis because of the significant errors in this region. The advantage of the structured light scanning technique over 3D-stereophotogrammetry is that a hand-held construction enables scanning from multiple angles, which may be beneficial for capturing the periorbital region. Also, the position of the patient is of importance when capturing the eyes and peri-orbit because a change in gaze direction may alter the amount of visible eyelid skin. Therefore, the frequently used natural head position (NHP) might not be sufficient for accurate 3D-scans. Additional measures, such as fixing the head, might be needed to limit these movement artefacts during scanning.

It would be of great value for clinical decision making to be able to perform reproducible 3D measurements of the area around the eye. This would enable objective evaluations of surgical treatments in the periorbital region, such as blepharoplasties. Thus, it is important that the 3D-reconstruction of this area is reproducible. Hence, we assessed the reproducibility of using the Artec Space Spider, a 3D-scanning technological device on the periorbital region, first when the head is in a natural unsupported position and second when supported with a frame.

## Material and methods

All the employees of the Department of Oral and Maxillofacial Surgery of the University Medical Center Groningen, the Netherlands, were invited to participate. Anyone who was healthy was eligible, of whom the first 15 volunteers were included. Volunteers were excluded if they had any history of epilepsy or facial deformities. The study was approved by the medical ethics committee of the University of Groningen and University Medical Center Groningen (study number METc2018/531). The study protocol was in accordance to institutional guidelines and the Declaration of Helsinki. Informed consent was obtained from all the participants prior to the study.

To test the reproducibility of periorbital 3D-scans, full face 3D-scans of the volunteers were captured at different time-points using the handheld Artec Space Spider scanning device (Artec 3D, Luxembourg). Data acquisition and processing was performed with Artec Studio Professional (version 13.0).

**Figure 1:**
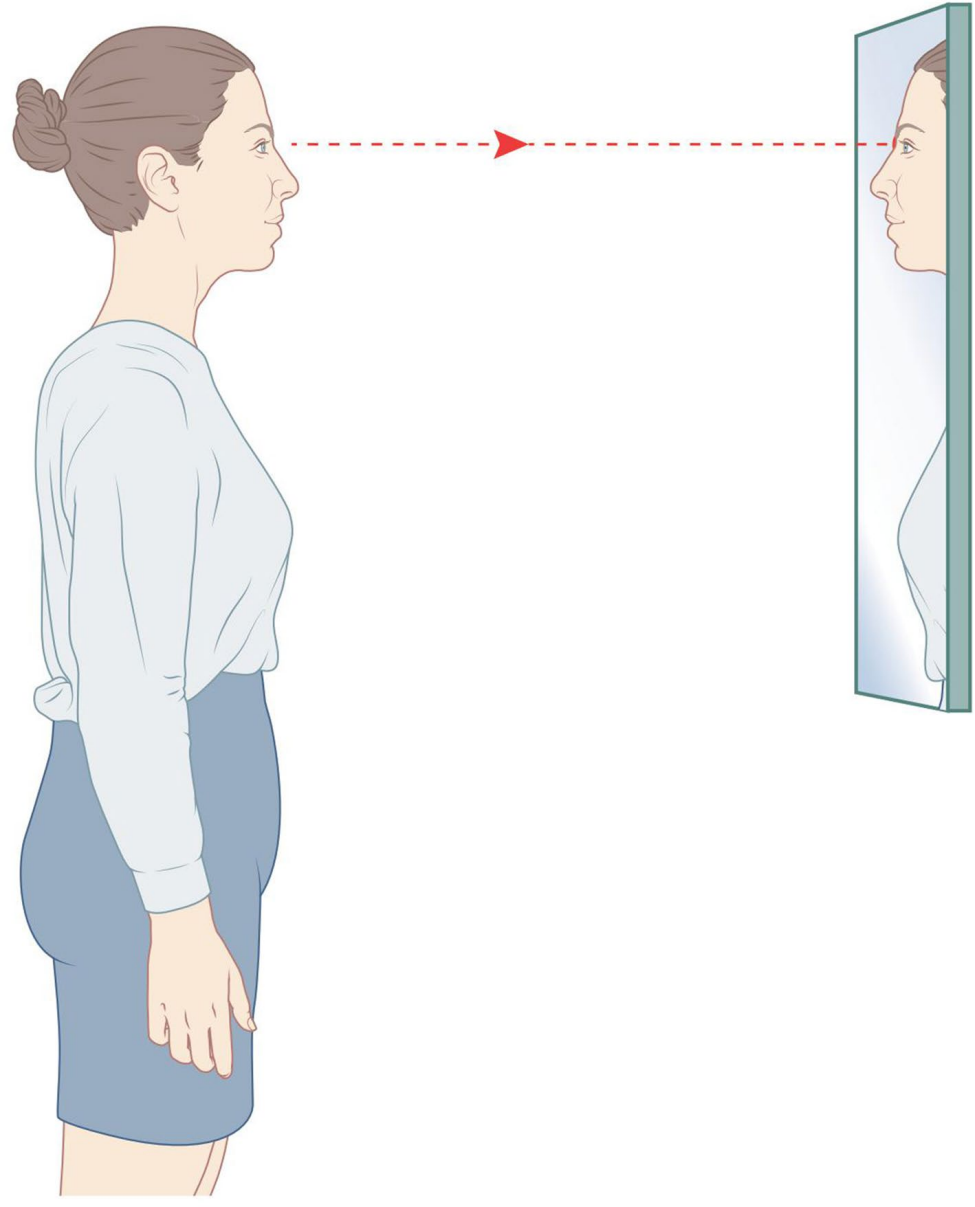
Natural head position. Created by Rogier Trompert Medical Art.

**Figure 2:**
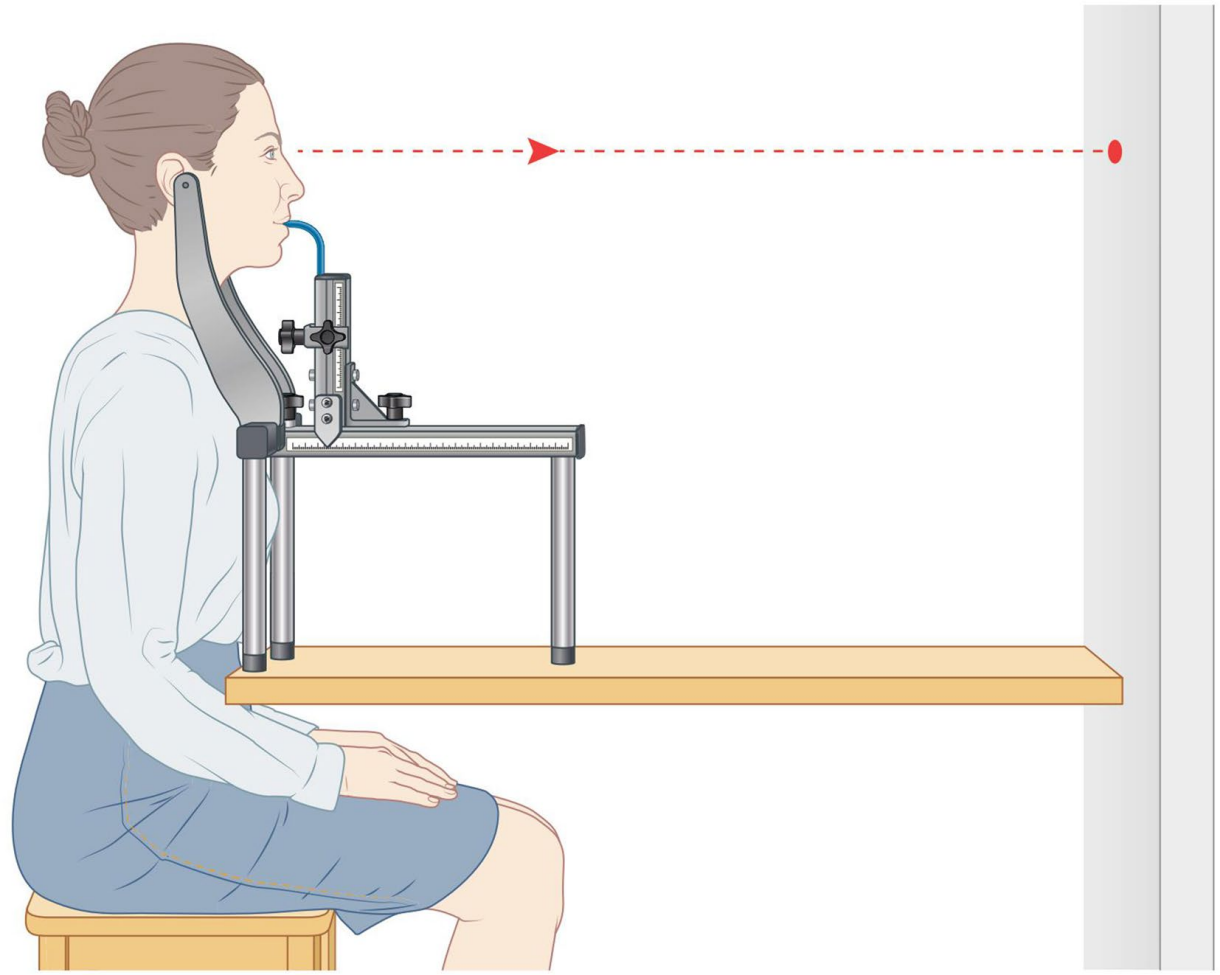
Fixation device. Created by Rogier Trompert Medical Art.

The volunteers’s head were scanned in two different positions in order to determine the most reproducible method, first in the Jakobsone et al.^15^ determined natural head position (Fig. 1) and second by fixating the head in a head frame designed for this purpose (Fig. 2). Fixation was achieved by placing earbuds, that were attached to the device by bars, in both of the participant’s ears. The bars were slightly curved in order not to interfere with the scanning surface of the cheeks. The bars could be moved away and towards each other, enabling optimal positioning of the head. The distance between the bars was recorded in millimetres. Next, a small mouthpiece was placed between the anterior teeth to restrict movements in the sagittal plane. The distance of the mouthpiece was also recorded in millimetres. The volunteers were asked to look at a mark on the wall during the 3D-scanning and the height of the whole fixation-device was based on the marking on the wall. The fixation device was made of metal to avoid bending and movement during the scanning procedure. The volunteers were asked not to wear any make-up.

### Scanning technique

Before scanning, the 3D-scanner was warmed up according to the recommendations of the manufacturer to achieve maximum accuracy. During the first scan (T1), to achieve a natural head position and habitual occlusion, the volunteers had to stand upright and were asked to swallow and keep their molars softly in occlusion, while looking at themselves (into their own eyes) in the mirror with their habitual facial expression^15^. The volunteers were then placed in the fixation device for the second scan (T2), while continuously looking with a habitual facial expression at a fixed point on the wall (at eye height). After this, another 3D-scan was made in the natural head position (T3) and then another with the head fixated in the frame (T4). One year later, the volunteer was scanned in the natural head position (T5) and then while fixated (T6), after identical instructions. Immediately after this, another 3D-scan of the head in the natural head position (T7) and with fixation (T8) was performed. This resulted in scanning every volunteer eight times.

The scanning after one year took place at the same time of day as the first scans in order to minimize the possibility of circadian volume shifts of the face. The volunteers were checked for changes in body weight, medication, medical condition and lifestyle. All the scans were made by the same trained investigator (MH) and were taken in the same room and under the same lighting conditions. Also, the 3D-analysis was done by a single investigator (AMLM).

To capture the upper face fully, each face was scanned in multiple passes, with significant overlap in the object coverage to allow for successful individual rigid scan alignment and registration. Different angles were used in a fluent movement in order to fully capture the whole periorbital area. Real-time fusion enabled visual control of completeness during and immediately after the scanning. A range indicator was available in Artec Studio 13.0 (Artec, Luxembourg) which visualized the distance between the scanner and the object. The working distance was always within 0.2 to 0.3 m.

### Data processing (Figs. 3 and 4)

**Figure 3:**
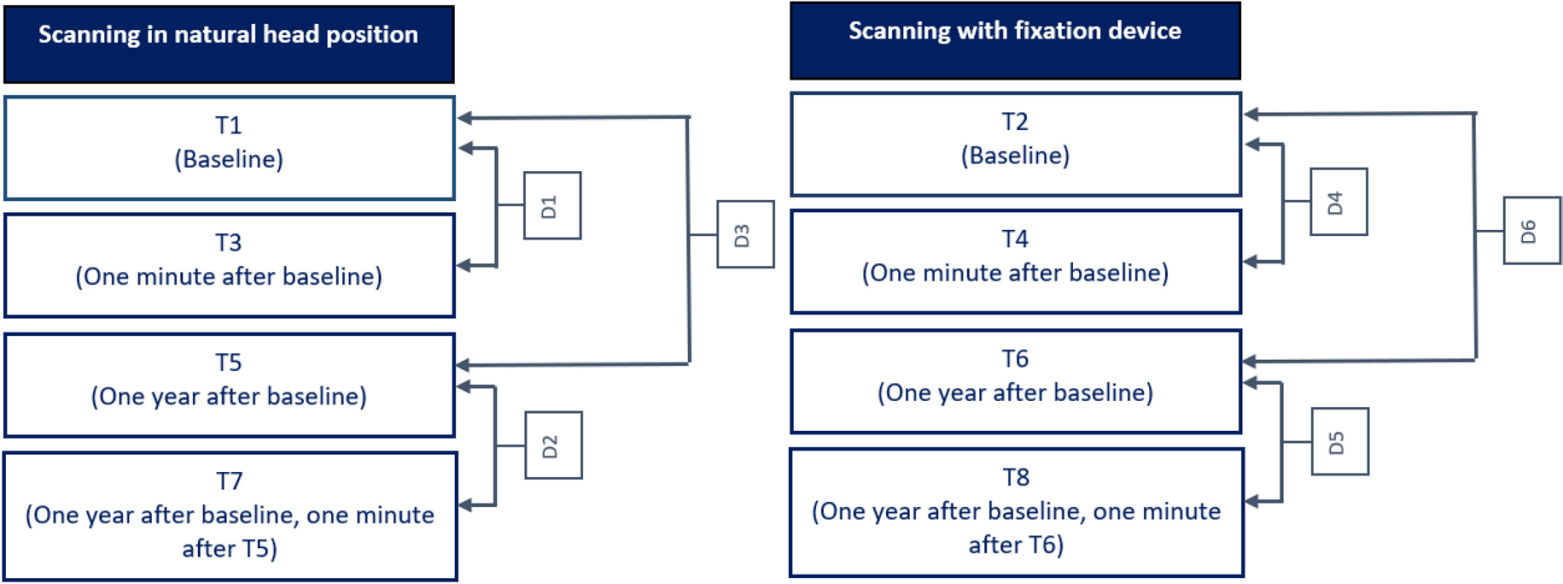
3D scanning and comparisons of deviations between 3D-scans. ‘D’ indicates the deviation of the 3D-scans between the different measurements.

**Figure 4:**
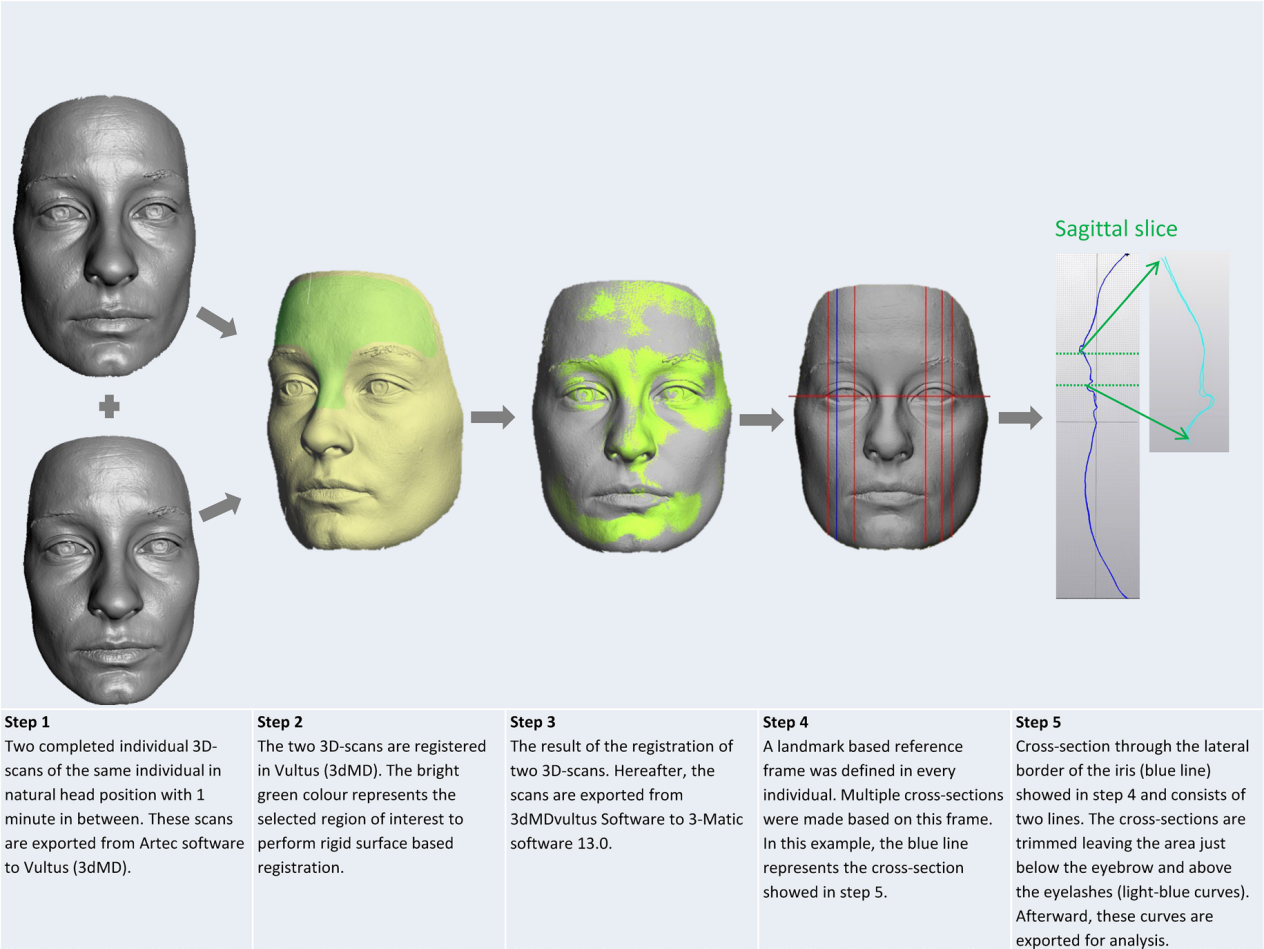
Data processing flowchart. All the depicted data-processing steps are from scanning one volunteer’s natural head position. Used software to create the images in this figure: Artec Studio version 13.0 (Artec, Luxembourg, http://www.artec3d.com), 3dMDvultus Software version 2.6.0.1 (3dMD LLC, USA, Atlanta, GA, http://www.3dmd.com) and 3-Matic software version 13.0 (Materialise, Leuven, Belgium, http://www.materialise.com).

The Artec Space Spider makes multiple image frames during acquisition. Afterwards, the Artec software processes the geometrical data of all the frames to calculate a 3D-model. During the acquisition time, movement artifacts can occur such as blinking of the eyes. Therefore, all the frames were first assessed by the investigator to eliminate scans in which the eyes were not fully open. Each completed scan was manually subjected to a serial registration procedure (Artec Studio 13.0). A fine, and thereafter global registration of all the selected frames in an individuals’ scan, was performed followed by visual inspection of the results. Any artifacts were removed using the ‘small object filter-function’ (to remove small surfaces unconnected to the main surface of the face) and ‘outlier removal’ (filtering algorithm to remove outliers; standard deviation multiplier 0.3), and the scans were fused using the Sharp Fusion-function (reconstruction of polygonal model, resolution 0.3, holes were not filled). These pre-processed 3D-images were exported as stereolithography files (STL-files) and stored for comparison purposes. The STL files of the multiple recordings (different time points, T1–T8) were imported into the 3dMDvultus Software (3dMD LLC, USA, Atlanta, GA) to perform a rigid surface based registration using the best fit surface-based method for every volunteer based on the selected region comprising the forehead (the area from below the hairline to above the eyebrows) and the upper nasal dorsum^1,16^. The quality of the alignment was assessed by visual inspection of the registered images and by providing the root mean square error (RMS-error) of the selected area (quality checks).

Following the surface based registration, the pre-processed 3D-images of the different time points (T1–T8) were exported from the 3dMDvultus Software to the 3-Matic software 13.0 (Materialise, Leuven, Belgium) for further analysis. In this stage, T1 was matched with T3 (natural head position comparison); and T2 was matched with T4 (head frame comparison). Also, T1 was matched with T5 (natural head after 1 year) and T2 was matched with T6 (head frame after 1 year). After that, T5 was matched with T7 (second natural head comparison with one minute in between) and T6 was matched with T8 (second head frame comparison with one minute in between). A landmark based reference frame was defined for every individual and was applied to all the scans after matching (T1–T8). Based on this reference frame, multiple cross-sections through the area of interest (peri-orbit) were obtained (see Fig. 4). The areas of interest (i.e., where the cross-sections were made) were the lateral canthus, the lateral border of the iris, and the medial border of the iris, on both eyes. After trimming the cross sections to the peri-orbital area only, leaving only two curves for each matched pair of 3D scans, a deviation analysis was performed. To determine the average deviation, i.e. the average difference in distance, (in millimetres) between the curves (cross-sections of T1–T8) the curves were exported to Matlab (The MathWorks, Inc., Natick, MA, USA). This function calculates the distance between the two curves by measuring the nearest opposing point of the counter parting curve, along the whole curves. These distance measurements served as a measure of the reproducibility of the 3D-images of the peri-orbital region made by the Artec Space Spider 3D-scanner.

The average deviations (D) were calculated per anatomical area as follows (Fig. 3):

- D1 = NHP scan at baseline (T1) versus NHP scan after 1 min (T3)
- D2 = NHP scan 1 year after baseline (T5) versus NHP scan 1 min after T5 (T7)
- D3 = NHP scan at baseline (T1) versus NHP scan one year after baseline (T5)
- D4 = fixed position scan at baseline (T2) versus fixated position scan after 1 min (T4)
- D5 = fixed position scan 1 year after baseline (T6) versus fixated position scan 1 min after T6 (T8)
- D6 = fixed position scan at baseline (T2) versus fixation position scan 1 year after baseline (T6)

Intra-tool reliability was assessed for the natural head position based on D1 and D2. Similarly, intra-tool reliability was assessed for the fixed head position based on D4 and D5. Inter-tool reliability was assessed for the natural head position and the fixed head position based on D1 and D4.

### Statistical analysis

The data was analysed with SPSS Statistics version 23.0 (IBM Corp., Armonk, NY, USA). Q–Q plots were used to determine the distribution of the data. When the data did not show a normal distribution, descriptive statistics in the form of median and interquartile ranges (IQR:Q3-Q1) were provided.

To assess the reproducibility of the data processing (see Fig. 4: flowchart data processing), all the steps were repeated for a randomly chosen cross-section of the natural head position and of the fixated head position, namely the lateral iris of the left eye. The data processing was carried out by the same researcher (AMLM) two months after the first data-processing round was completed. The limits of agreement between the first processing round and the second processing round were shown in a Bland Altman plot and reliability was calculated by estimating the intraclass correlation (ICC; two-way mixed effects model, single measurement, absolute agreement) and 95% confidence interval (CI).

Plots were made in order to investigate possible systematic differences between the minute-interval scanning moments for the natural head position (D1 vs D2) and the fixated head position (D4 vs D5). A red intermittent line was marked to show the 1 mm deviation cut-off, which is commonly considered to be the clinical acceptable deviation^6,17–19^. Bland Altman plots showed the differences in the deviations were normally distributed, as assessed from the Q-Q plots, and therefore the assumptions were proven to be valid.

The ICC values were interpreted as follows: 0.00–0.20, poor; 0.20–0.40, fair; 0.40–0.60, moderate; 0.60–0.80, good; 0.80–1.00, excellent^20^. When interpreting the results for research purposes (comparing groups), the ICC should be at least 0.70, while for clinical practice, the ICC should at least be 0.90^21^.

The test–retest reliability (reliability of replications of the minute-interval 3D-deviation measurements; D1 and D2, D4 and D5) was evaluated by calculating the intraclass correlation coefficient (ICC) and confidence intervals. The average deviations between the 3D-scans when performing consecutive 3D-scans (one minute interval; n = 15; D1 and D4) and when repeating this one year later (second one minute-interval; n = 14; D2 and D5) were calculated using Matlab. Two comparisons were made per scanning technique, NHP and fixated head position, in order to assess the ICC between both minute-interval scanning-events.

## Results

A total of 15 healthy volunteers participated in this study with a median age of 47 years (range: 30–63 years, IQR: 42–58 years). Of these 15 volunteers, 10 were female (67%). After one year, no changes were reported by the participants in medication, medical history and lifestyle, but weight changes had occurred by a median + 1 kg (range: − 2 kg to + 7 kg, IQR: 0 kg to 2 kg). One male volunteer was excluded from the year follow-up due to personal circumstances and one female because of pregnancy.

### Reproducibility of 3D processing

**Figure 5:**
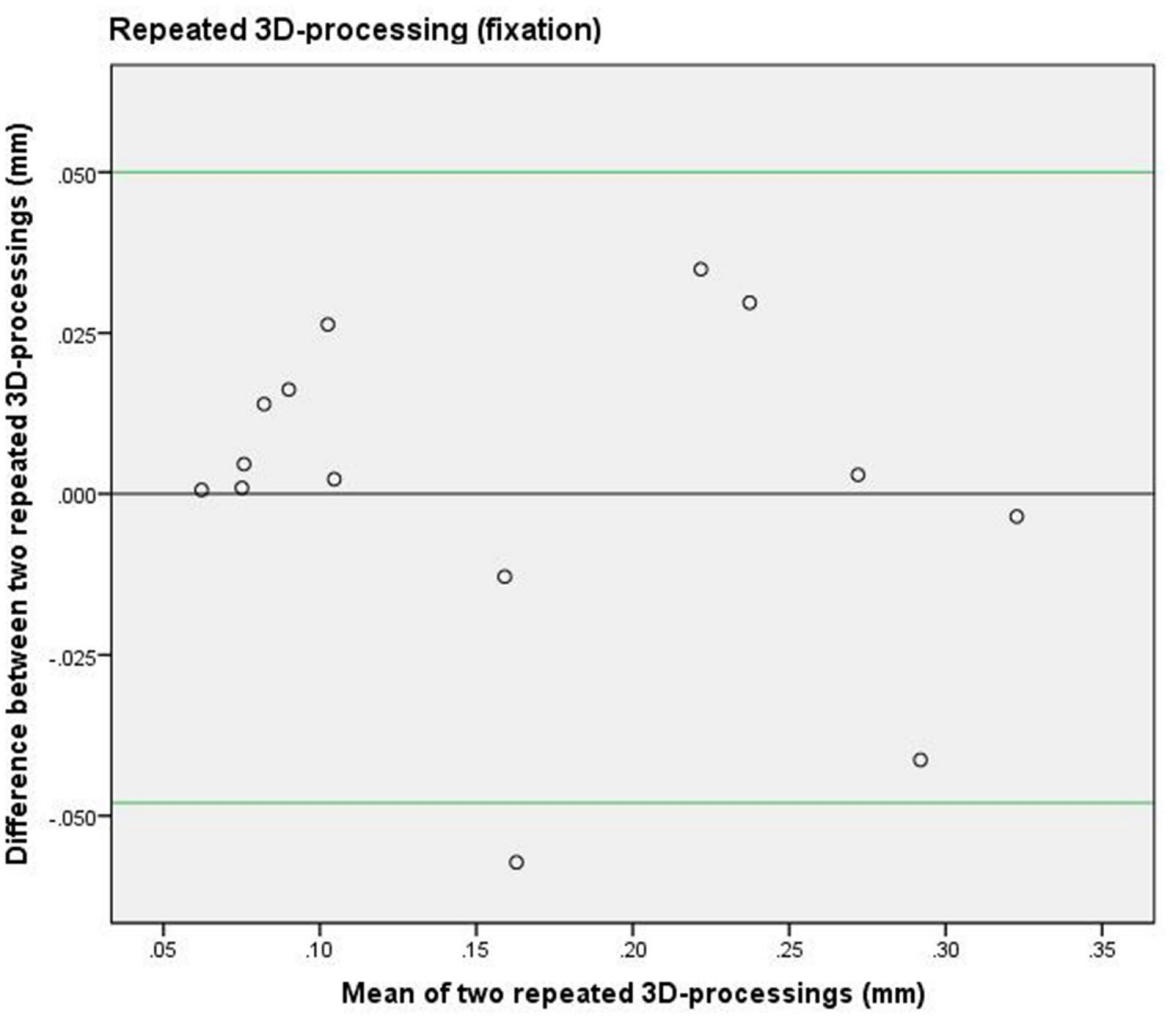
Bland Altman plot of repeated data processing (fixation). The 3D-data processing was repeated for the second minute-interval scans and comprised the cross section of the lateral iris of the left eye during fixation. The green lines represent the upper and lower limits of agreement between the two 3D-data processing rounds.

**Figure 6:**
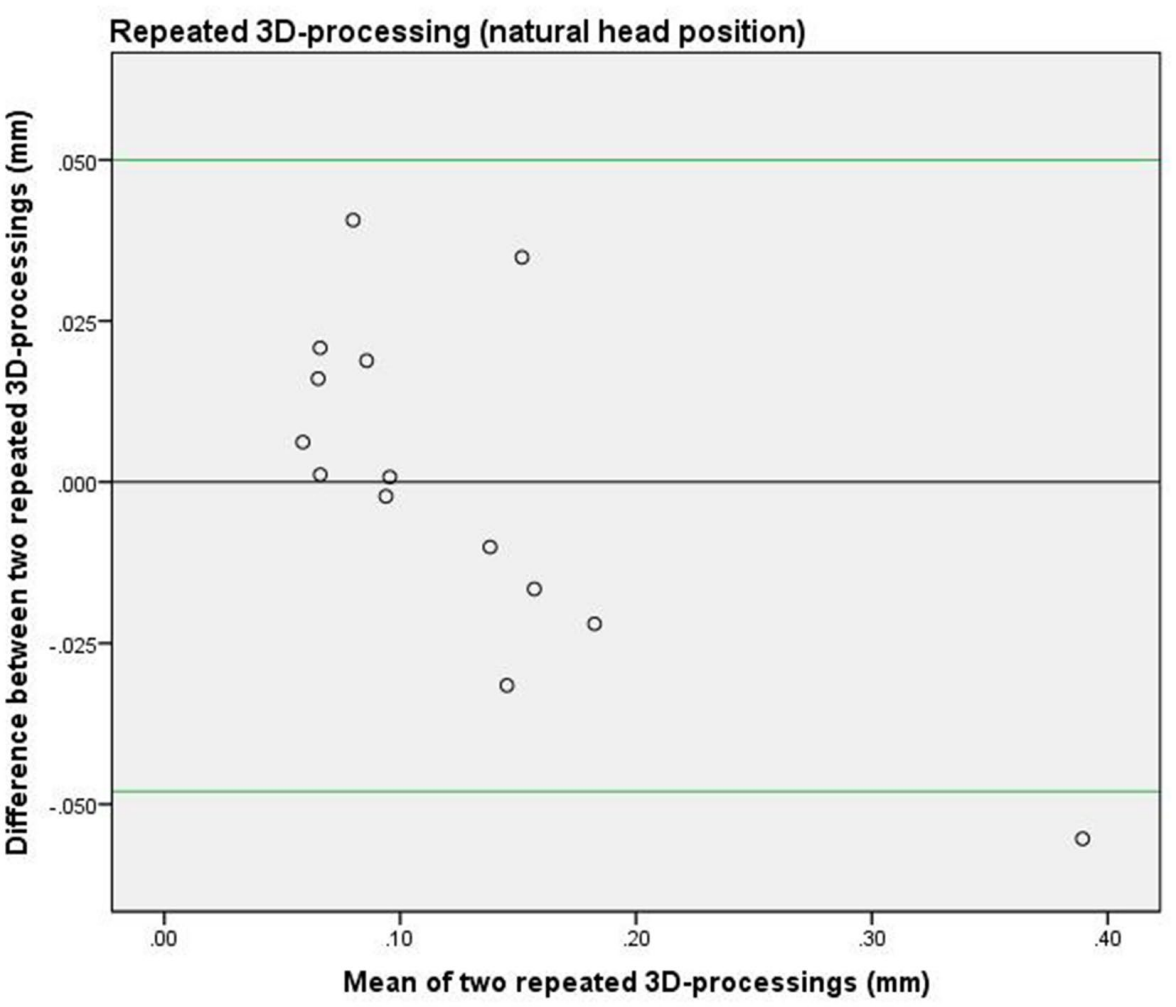
Bland Altman plot of repeated data processing (NHP). The 3D-data processing was repeated for the second minute-interval scans of the natural head position and comprised the cross section of the lateral iris of the left eye. The green lines represent the upper and lower limits of agreement between the two processing rounds.

The 3D-processing was repeated to assess the reproducibility between the facial models of the two processing-rounds. Two Bland Altman plots (fixation and NHP) were drawn (Figs. 5 and 6) and the agreement limits were between − 0.05 and 0.05 mm.

The ICC of the same cross-section on the 3D NHP scans was 0.96 (95% CI: 0.87–0.99) and on the fixation device scans it was 0.97 (95% CI: 0.90–0.99).

### Reproducibility of 3D-scans

The descriptive statistics of the cross-section curves are given in Table 1. On comparing the one minute interval 3D-scans of the area between the eyelashes and eyebrows (D1, D4), the median of the average deviations between the cross-sections ranged from 0.17 to 0.21 mm. After performing the one minute-interval scans one year later (D2, D5), the median differences were 0.10–0.11 mm. All the patients’ one minute-interval scanning deviations and differences between the intervals were less than 1 mm.

**Table 1:** Descriptive statistics and intra-tool reliability (ICC and 95% confidence intervals).

	First minute-interval scanning (baseline)	Second minute-interval scanning	Year-interval scanning	First minute interval deviations (baseline) vs second minute interval deviations	First minute interval deviations vs second minute interval deviations
	Average deviation between two subsequent scans (median and Q1–Q3 in mm)	Average deviation between two subsequent scans (median and Q1–Q3 in mm)	Average deviation between two scans with one year in between (median and Q1–Q3 in mm)	ICC and 95% confidence interval	Number of patients with a difference of ≥ 1 mm between D1 and D2
Natural head position	D1	D2	D3	D1 vs D2	D1 vs D2
OD lateral canthus	0.14 (0.11–0.34)	0.10 (0.09–0.13)	0.44 (0.33–0.75)	0.07 (− 0.27 to 0.50)	0
OD lateral iris	0.15 (0.12–0.32)	0.11 (0.09–0.16)	0.52 (0.34–0.72)	0 (0–0.41)	0
OD medial iris	0.16 (0.12–0.29)	0.12 (0.07–0.14)	0.43 (0.32–0.69)	0 (0–0.33)	0
OS lateral canthus	0.21 (0.17–0.33)	0.12 (0.08–0.15)	0.36 (0.29–0.58)	0.01 (0–0.40)	0
OS lateral iris	0.22 (0.13–0.33)	0.10 (0.08–0.15)	0.38 (0.28–0.69)	0.09 (0–0.50)	0
OS medial iris	0.15 (0.10–0.32)	0.11 (0.08–0.13)	0.43 (0.24–0.60)	0.05 (0–0.47)	0
Average of all cross-sections combined (in mm)	0.17	0.11	0.43		

Fixated head position	D4	D5	D6	D4 vs D5	D4 vs D5
OD lateral canthus	0.16 (0.12–0.27)	0.09 (0.08–0.11))	0.48 (0.31–0.75)	0.17 (0–0.62)	0
OD lateral iris	0.19 (0.14–0.28)	0.09 (0.08–0.11)	0.48 (0.26–0.69)	0 (0–0.35)	0
OD medial iris	0.17 (0.14–0.29)	0.08 (0.07–0.10)	0.39 (0.29–0.59)	0 (0–0.43)	0
OS lateral canthus	0.26 (0.20–0.31)	0.10 (0.08–0.23)	0.48 (0.38–0.84)	0.22 (0–0.64)	0
OS lateral iris	0.27 (0.16–0.41)	0.13 (0.09–0.26)	0.44 (0.31–0.68)	0.32 (0–0.72)	0
OS medial iris	0.19 (0.12–0.37)	0.10 (0.08–0.18)	0.46 (0.26–0.72)	0 (0–0.32)	0
Average of all cross-sections combined (in mm)	0.21	0.10	0.46		

1 min-interval versus 1 year-interval: median of average deviations between the two curves from the natural head position (NHP) and the two curves from the fixated head position (in mm). The shown intraclass correlation coefficients (ICC) and 95% confidence intervals are all < 0.9.

*OD* oculus dexter (right eye), *OS* oculus sinister (left eye).

On comparing the baseline-scans with those after one year (D3, D6), the median deviations between the cross-sections had increased to 0.43–0.46 mm, but all of them remained below the 1 mm (clinical) limit.

**Figure 7:**
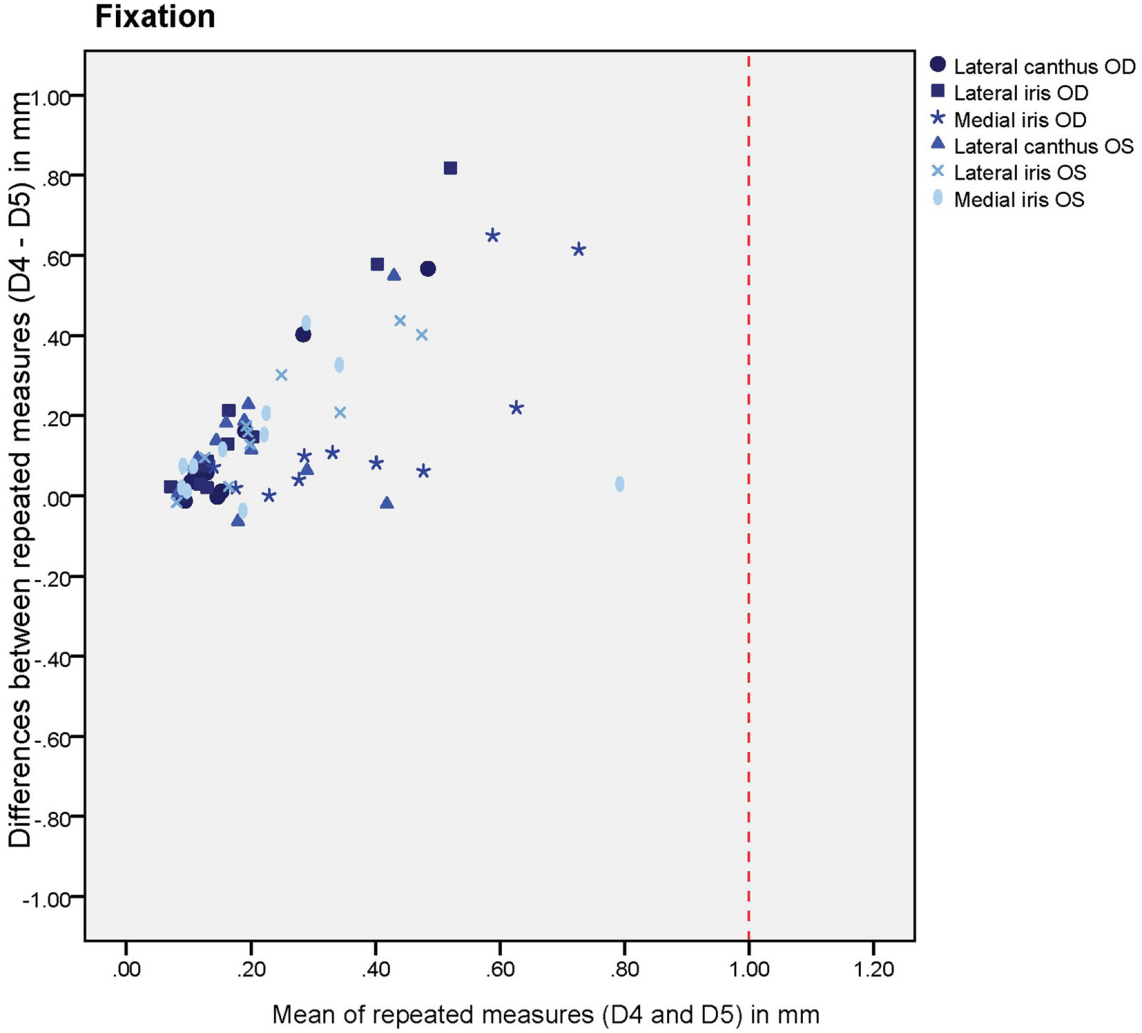
Systematic differences of different anatomical sites after scanning the head in a fixed position. Differences between the first minute-interval deviations and second minute-interval deviations were computed by the following subtraction: first minute-interval deviations (D4) – second minute-interval deviations (D5). *OD* oculus dexter (right eye), *OS* oculus sinister (left eye). The red intermittent line shows the clinical 1 mm mean deviation cut-offs in subsequent scans. All the mean deviations were below this cut-off.

**Figure 8:**
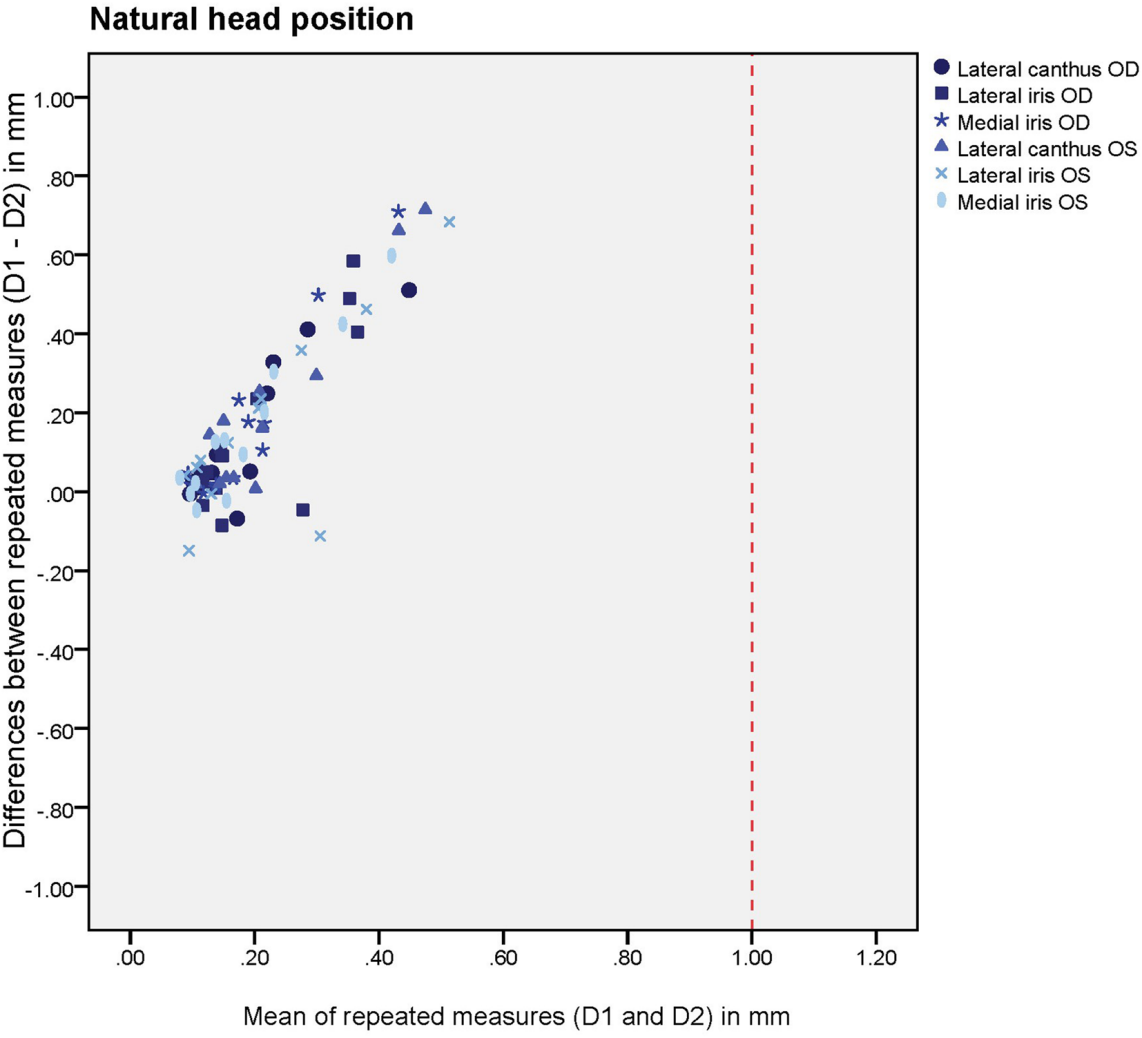
Systematic differences of different anatomical sites after scanning the head in a natural position. Differences between the first minute-interval deviations and second minute-interval deviations were computed by the following subtraction: first minute-interval deviations (D1) – second minute-interval deviations (D2). *OD* oculus dexter (right eye), *OS *oculus sinister (left eye). The red intermittent line shows the clinical 1 mm mean deviation cut-offs in subsequent scans. All the mean deviations were below this cut-off.

Figures 7 and 8 show the systematic differences of the fixed position and NHP between the first minute-interval deviations (D1, D4) and the second minute-interval deviations (D2, D5). The graphs show that all the mean deviations were below 1 mm. When the mean deviations were small, the differences between the repeated measurements were also small. However, when the mean deviations were greater, the differences between the repeated measurements were greater and therefore less reliable, even though they were always within the 1 mm criterion. Also, the differences between the repeated measurements were predominantly positive. Therefore, the baseline deviations were greater than those of the second interval scans. The ICC between the first minute-interval scanning deviation (D1, D4) and second minute-interval scanning deviation (D2, D5) was < 0.7 (Table 1).

### Comparison of scanning methods: NHP and fixation

The median of the average differences between the NHP and fixation was small (Table 1). Figures 5 and 6 show that the pattern of the systematic differences between scanning the natural head position and in the fixated position were comparable.

### Evaluation of variations in cross-section locations

Figures 5 and 6 show that the systematic differences between the cross-section locations follow a similar pattern. However, the cross section of the medial iris of the right eye, when the head is in a fixated position, shows the most favourable pattern (medium blue asterisks in Fig. 8).

### Quality checks

The quality of the alignment during the data processing was assessed by inspecting the registered images visually and by providing the root mean square error (RMS-error) of the selected area. Visual inspection did not reveal any incorrect registrations. The median RMS-error of the registration during the first one minute-interval (T1 and T3) was 0.13 (range 0.05–0.81, Q1–Q3 0.07–0.27) for the natural head position and for the fixated position (T2 and T4) it was 0.11 (range 0.01–0.39; Q1–Q3 0.06–0.17). When performing the one minute-interval registrations a year later (T5 and T7, T6 and T8), the median RMS-error for the natural head position was 0.04 (range 0.02–0.18; Q1–Q3 0.03–0.06 and for the fixated head it was also 0.04 (range 0.03–0.12; Q1–Q3 0.03–0.08).

The one year interval 3D-scan registrations provided an RMS-error of 0.17 (range 0.08–0.30; Q1–Q3 0.10–0.22) for the natural head position (T1 and T5) and 0.15 (range 0.11–0.37; Q1-Q3 0.14–0.19) for the fixated head (T2 and T6).

## Discussion

For objective evaluation of surgical treatment in the periorbital region 3D-imaging of this area should be reproducible. To our knowledge, this is the first study to specifically assess the reproducibility of 3D-imaging of the periorbital area between the upper eye lashes and the brow. There is a trend to preserve and redistribute volume during aesthetic periorbital surgery. Hence, the three-dimensional periorbital imaging proposed in this study could provide more insight into the effects of such procedures.

In the literature, the region of the eyes is known to be accompanied by significant errors in 3D-imaging and is often excluded from analyses^2, 7, 22^. This is due to difficulty in capturing the eyes correctly because the light pattern used to reconstruct a 3D-photograph interferes with the light reflection in the eyes’ lenses whereby the lenses appear to be concave instead of convex. Also, the position of the eyes in relation to the supraorbital ridge and eyebrows might influence 3D-scanning of this area. Deep-set eyes with prominent supraorbital ridges, which is commonly seen male eye-and-eyebrow complex^23^, might be more difficult to capture in 3D images. In addition, differences in facial expression or posture during separate scans can also result in registration error^5^, and the eyes are difficult to capture due to frequent blinking movements.

The current study focused on minimizing the factors hindering the assessment of 3D-images of the periorbital area and so areas like the cornea, eyelashes and eyebrows were not included in the final analysis. On applying our technique, the median average deviation between two subsequent scans was approximately 0.1–0.2 mm, significantly below the 1 mm cut-off, which is proposed as an acceptable deviation in clinical practice. Yet, although the 3D-processing method showed excellent reproducibility, scanning the individuals repeatedly resulted in lower reproducibility. However, when assessing the systematic differences between the scans taken of each subject, these differences were predominantly positive. This means that the deviating differences seen at baseline had reduced further on scanning the same person one year later, implying a positive scanning learning curve, which explains the low ICC. Additionally, slight changes in muscle tone or expression between the scanning events can also cause small differences in 3D-scans. We have to also take the system and 3D-processing errors into account. The latter was found to be between -0.05 mm and 0.05 mm in this study but, according to the manufacturer, the system’s error is also 0.05 mm (the 3D-point accuracy of the scanner is 0.05 mm or less). Irrespective of this, some uncertainties might be present regarding the accuracy of the scanner, such as a possible cumulation of error due to the iterative mesh build of the 3D-points and one study found that the scanner does not meet the declared precision^13^. Further research has to be done to elucidate this issue. Nevertheless, the effects of a blepharoplasty, for example, will result in alterations of several millimetres, which can thus be captured adequately with the technique.

The median of the average difference between the scans made at baseline and after one year was approximately 0.45 mm. The scans were repeated after one year because, in clinical practice stable results are usually obtained before the end of the first year after surgery. Presumably, natural changes such as weight ^9,24,25^, tiredness^26^ and aging^27^ occur in the face, so it could be justified that, when applying a very sensitive scanning method, there will be differences between the baseline and the measurements taken one year apart. We assessed a group of 15 participants to evaluate repeatability in a group with a variety of age-categories and gender because this reflects daily clinical practice.

The median of the average deviations found in our study for the orbital region are in the same order of magnitude as described in the literature for the total face; in the literature, 100 3D-images of one individual had a RMS-error of 0.36 mm after 6 weeks for the overall face^1^, yet the RMS-error for the periorbital area was 0.38 mm (1.02 mm 95th percentile). Unfortunately, it is unclear whether this error was calculated from subsequent scans or from comparing those with 6 weeks in between. Johnston et al.^28^ found a deviation of 0.74 mm for the overall face at rest when assessing the reproducibility, but the upper eyelids’ region was not an included landmark. Kau et al.^29^ found an average mean deviation of 0.25 mm, with a maximum of 0.49 mm, in adults when scanning subsequently using a Minolta Vivid 900 laser scanner. Ma et al.^30^ scanned participants at baseline and again after 1 day, 3 days, 1 week and 3 weeks and used a structured light system. The mean deviation of the whole 3D facial image was 0.20 mm and the maximum deviation was 0.32 mm. The latter is quite small compared to the maximum deviations in our study but, in their article, the 3D facial image did not capture the upper eyelids completely.

When assessing the periorbital area scans, we tried to eliminate any possible hindering factors, such difficulties to capture the eyelids completely. The largest variations found by other authors who collected data with a 3D-stereophotogrammetric camera setup were in the periorbital area^7^. The disadvantages of such a setup is that the camera is not mobile and that certain areas of the face are not imaged completely due to a fixed camera orientation and focus point of the camera. In contrast, the Artec Space (Artec 3D, Luxembourg) is a structured light hand-held scanning device. The technique combines structured light 3D-scanning (blue LED), for assessing the shape, with an image-based approach (white flash light) to add supplementary shape information and colour textures. Thus, we could capture the eyelids completely with this device. Also, to eliminate variations in the amount of eyelid exposed during scanning caused by a changed point of gaze, we compared the effect of using a fixation-device with the frequently used natural head position. In contrast to our expectations, median deviations and the comparable patterns in the plots of the scanning-deviations were comparable for both methods. Although no scanning method seems to be superior to the other, the natural head position is more comfortable for the participant and therefore preferable.

To eliminate movement artifacts caused by blinking, all the separate frames of the 3D-scans were screened. So, of the approximately 400 frames of each scan, a maximum of 5 frames had to be excluded due to blinking. Within the anatomic units, we used the most stable regions (i.e., the forehead and nose^1^) to be registered with each other. The region of interest, in this case the periorbital region, was intentionally excluded from the registration process. It is important that, for clinical purposes, the registration process is accurate, with less than 1 mm variation^31^. In the current study, all the RMS-errors of the registered areas were all below this range and registrations were therefore considered successful. After establishing a reliable registration, further analyses were carried out of the area between the upper eyelashes and below the eyebrow by evaluating the cross-section curves of this area. The cross-sections were considered to be stable and clearly definable and were therefore chosen to provide information on the areas of interest.

The scanning method itself has a few disadvantages. First, the used structured light system needs to be operated by a trained person. Although our 3D-scans were made by a trained operator, a learning curve was still observed. Furthermore, the time needed to capture a 3D-image of a face with the Artec Space Spider is longer than when using stereophotogrammetry. In theory, the longer a 3D-scan takes, the more artifacts can be caused by the subject moving. Another possible disadvantage is that the strobing structured light flashes might make it more difficult for participants to relax their eyes fully, although this was not observed here. Also, the moving scanner might distract the patient from gazing continuously at a fixed point throughout the scanning period.

The stereophotogrammetry 3dMD scanner is the most widely used scanning system for obtaining 3D scans of the face surface. Several hand-held scanners have been studied and compared to the 3dMD; the Artec EVA was shown to be less accurate than the 3dMD system^2–4^. However, the Artec Space Spider has better 3D-point accuracy, compared to the Artec Eva and 3dMD-sytem, based on scanning geometrically stable reference bodies^13^. Winkler et al.^32^ assessed the trueness and precision of scanners intended for scanning smaller areas (i.e., intraoral scanners), including the Artec Space Spider, and concluded that the latter was superior to the intraoral scanners. The high resolution makes it even possible to capture fingerprints accurately^33^. Nevertheless, further research has to be done with patients to compare the widely used 3dMD-system with the Artec Space Spider scanner.

## Conclusion

Scanning with a hand-held 3D-scanning device (Artec Space Spider) is a promising tool to assess changes in the periorbital region following surgical treatment since the median deviations are well below the clinically accepted 1 mm measuring error, for both the natural head and fixated positions.

## Data Availability

The dataset generated during and/or analysed during the current study are available from the corresponding author on reasonable request.
